# Different forms of glycine- and GABA_A_-receptor mediated inhibitory synaptic transmission in mouse superficial and deep dorsal horn neurons

**DOI:** 10.1186/1744-8069-5-65

**Published:** 2009-11-18

**Authors:** Wayne B Anderson, Brett A Graham, Natalie J Beveridge, Paul A Tooney, Alan M Brichta, Robert J Callister

**Affiliations:** 1 School of Biomedical Sciences and Pharmacy, The University of Newcastle and Hunter Medical Research Institute, University Drive, Callaghan, NSW 2308, Australia

## Abstract

**Background:**

Neurons in superficial (SDH) and deep (DDH) laminae of the spinal cord dorsal horn receive sensory information from skin, muscle, joints and viscera. In both regions, glycine- (GlyR) and GABA_A_-receptors (GABA_A_Rs) contribute to fast synaptic inhibition. For rat, several types of GABA_A_R coexist in the two regions and each receptor type provides different contributions to inhibitory tone. Recent work in mouse has discovered an additional type of GlyR, (containing alpha 3 subunits) in the SDH. The contribution of differing forms of the GlyR to sensory processing in SDH and DDH is not understood.

**Methods and Results:**

Here we compare fast inhibitory synaptic transmission in mouse (P17-37) SDH and DDH using patch-clamp electrophysiology in transverse spinal cord slices (L3-L5 segments, 23°C). GlyR-mediated mIPSCs were detected in 74% (25/34) and 94% (25/27) of SDH and DDH neurons, respectively. In contrast, GABA_A_R-mediated mIPSCs were detected in virtually all neurons in both regions (93%, 14/15 and 100%, 18/18). Several Gly- and GABA_A_R properties also differed in SDH vs. DDH. GlyR-mediated mIPSC amplitude was smaller (37.1 ± 3.9 vs. 64.7 ± 5.0 pA; n = 25 each), decay time was slower (8.5 ± 0.8 vs. 5.5 ± 0.3 ms), and frequency was lower (0.15 ± 0.03 vs. 0.72 ± 0.13 Hz) in SDH vs. DDH neurons. In contrast, GABA_A_R-mediated mIPSCs had similar amplitudes (25.6 ± 2.4, n = 14 vs. 25. ± 2.0 pA, n = 18) and frequencies (0.21 ± 0.08 vs. 0.18 ± 0.04 Hz) in both regions; however, decay times were slower (23.0 ± 3.2 vs. 18.9 ± 1.8 ms) in SDH neurons. Mean single channel conductance underlying mIPSCs was identical for GlyRs (54.3 ± 1.6 pS, n = 11 vs. 55.7 ± 1.8, n = 8) and GABA_A_Rs (22.7 ± 1.7 pS, n = 10 vs. 22.4 ± 2.0 pS, n = 11) in both regions. We also tested whether the synthetic endocanabinoid, methandamide (methAEA), had direct effects on Gly- and GABA_A_Rs in each spinal cord region. MethAEA (5 μM) reduced GlyR-mediated mIPSC frequency in SDH and DDH, but did not affect other properties. Similar results were observed for GABA_A_R mediated mIPSCs, however, rise time was slowed by methAEA in SDH neurons.

**Conclusion:**

Together these data show that Gly- and GABA_A_Rs with clearly differing physiological properties and cannabinoid-sensitivity contribute to fast synaptic inhibition in mouse SDH and DDH.

## Background

The superficial and deep laminae of the spinal cord dorsal horn, termed SDH and DDH respectively, are important sites for processing sensory information arising in skin, muscle, joints and viscera [1,2]. This information arrives in the dorsal horn via primary afferents, which have specific termination patterns in SDH (laminae I-II) and DDH (laminae IV-VI) depending on their axon diameter, sensory modality and peripheral origin [3-5]. The SDH receives inputs predominately from small diameter Aδ and C-fibres carrying nociceptive, thermal, itch, and innocuous tactile information [6-11]. In contrast, the DDH receives inputs predominately from larger Aβ myelinated fibres carrying tactile information [3,12,13].

As with primary afferent inputs, the outputs of the SDH and DDH, which are conveyed by projection neurons, also differ. Projections from the SDH terminate mostly in brainstem and midbrain centres such as the parabrachial nuclei and periaqueductal grey [14,15]. In contrast, projections from the DDH terminate mainly in the thalamus [16,17]. Recent evidence also suggests intrinsic synaptic connectivity differs in the two regions. For example, paired recordings in SDH neurons show a modular pattern of synaptic linkages connecting a restricted number of neuron types [18,19]. Similar recordings in the DDH suggest a more extensive synaptic connectivity among neuron classes [20].

These recent data suggest synaptic processing mechanisms differ in the SDH and DDH. Indeed, some aspects of fast inhibitory synaptic transmission, mediated by GABA_A _and glycine receptors (GlyRs) differ in the SDH and DDH. For example, c-Fos expression differs in the SDH and DDH after blockade of tonic inhibition with specific GABA_A _and GlyR antagonists [21] and an unusual form of the GlyR, containing alpha 3-subunits, is confined to the SDH of the mouse spinal cord [22]. These data suggest the two receptors play differing roles in setting inhibitory tone and subsequent processing of sensory information in the two regions.

The type-one cannabinoid receptor (CB_1_R), which has long been known to be involved in analgesia [23,24], is also expressed at different levels in the SDH versus DDH [25]. The CB_1_R appears to be more highly expressed in the SDH, consistent with the analgesic action of cannabinoids. Several mechanisms have been proposed for the analgesic action of cannabinoids including modulation of glutamatergic [26,27], noradrenergic [28] and opioidergic systems [29,30]. In addition to these CB_1_R mediated effects, several reports have suggested cannabinoids also act directly on GlyRs [31,32]. One study reported attenuation of glycinergic currents by cannabinoids in isolated hippocampal and Purkinje neurons [32], whereas another has reported cannabinoids potentiate glycine-activated currents in isolated ventral tegmental area neurons and in recombinant GyRs [31]. No studies have investigated the effects of cannabinoids on "native", synaptically located GlyRs on SDH or DDH neurons.

In this paper, we first characterize synaptically-mediated miniature inhibitory postsynaptic currents (mIPSCs) in both the SDH and DDH of the mouse spinal cord using patch clamp techniques. We then test whether the synthetic endocannabinoid, methanadamide (methAEA), has direct effects on synaptically located Gly- and GABA_A_Rs in the SDH and DDH. Finally, we use real-time PCR (qPCR) to quantify and compare subunit expression of Gly- and GABA_A_Rs, and the CB_1_R in the SDH and DDH.

## Methods

### Tissue preparation

All experimental procedures were approved by the University of Newcastle Animal Care and Ethics Committee. Mice (C57/Bl6; both sexes, aged 17-37 days) were anaesthetized with Ketamine (100 mg kg^-1 ^i.p.) and decapitated. The vertebral column (~T5 - S1) was isolated and immersed in ice-cold oxygenated sucrose substituted artificial cerebro-spinal fluid (S-ACSF). This solution contained (in mM): 250 sucrose, 25 NaHCO_3_, 10 glucose, 2.5 KCl, 1 NaH_2_PO_4_, 1 MgCl_2 _and 2.5 CaCl_2 _and was bubbled with Carbogen (95% O_2 _and 5% CO_2_). The lumbar spinal cord (L1 - L6) was removed, placed against a Styrofoam support block, and glued (rostral side down) to a cutting platform with cyanoacrylate glue (Loctite 454, Loctite, Caringbah, Australia). The cutting platform was then transferred to a cutting chamber, filled with ice-cold S-ACSF, and transverse slices (300 μm-thick) were obtained from the L3-L5 segments using a vibratome (Leica Microsystems, Wetzlar, Germany). Slices were transferred to a storage chamber containing Carbogen-bubbled artificial cerebro-spinal fluid (ACSF; 118 mM NaCl substituted for sucrose in S-ACSF) and allowed to equilibrate for 1 h before electrophysiological recording.

### Electrophysiology

Individual slices were transferred to a recording chamber (bath volume 0.4 ml) and continually perfused (exchange rate 4-6 bath volumes/min) with bubbled ACSF. Neurons were visualized in spinal cord slices using infrared differential interference contrast optics (IR-DIC) and a Hamamatsu charge coupled device camera (Model C-2400-79H, Hamamatsu City, Japan) linked to a video monitor. Under IR-DIC lamina II appears as a translucent band that clearly delineates the lamina II-III border in mice older than six days (P6) [33]. All SDH recordings were made between this border and the dorsal white matter. We defined the DDH as the grey matter dorsal to the central canal, and more than 100 μm ventral to lamina II. All our DDH recordings were made within these boundaries. Patch pipettes (3-4 MΩ resistance), made from borosilicate glass (1.5 mm O.D; PG150T-15; Harvard Apparatus, UK) were filled with an internal solution containing (in mM): 130 CsCl, 10 HEPES, 10 EGTA, 1 MgCl_2_, 2 ATP and 0.3 GTP (pH adjusted to 7.35 with 1 M CsOH). Under these recording conditions the reversal potential for chloride ions is 0 mV: thus, at a holding potential of -70 mV all chloride-mediated inhibitory currents are inward. Whole-cell voltage-clamp recordings were made at room temperature (22 - 24°C) from SDH and DDH neurons. After obtaining the whole-cell recording configuration, series resistance and neuronal input resistance were assessed according to the response to a 5 mV hyperpolarizing step (average of 20 repetitions, holding potential -70 mV). These values were monitored at the beginning and end of each recording session, and data were rejected if values changed by > 20%. Series resistance (< 20 MΩ) was uncompensated in all experiments. All synaptic currents were recorded at a holding potential of -70 mV using an Axopatch 200B amplifier (Molecular Devices, Sunnyvale, CA, USA). Signals were filtered at 2 kHz and digitized on-line at 10 kHz via an Instrutech ITC-16i A/D board (Instrutech, Long Island, NY, USA). Data were stored on a Macintosh G4 computer and analysed offline using Axograph v4.6 software (Molecular Devices, Sunnyvale, CA, USA).

### Experimental protocols

**Figure 1:**
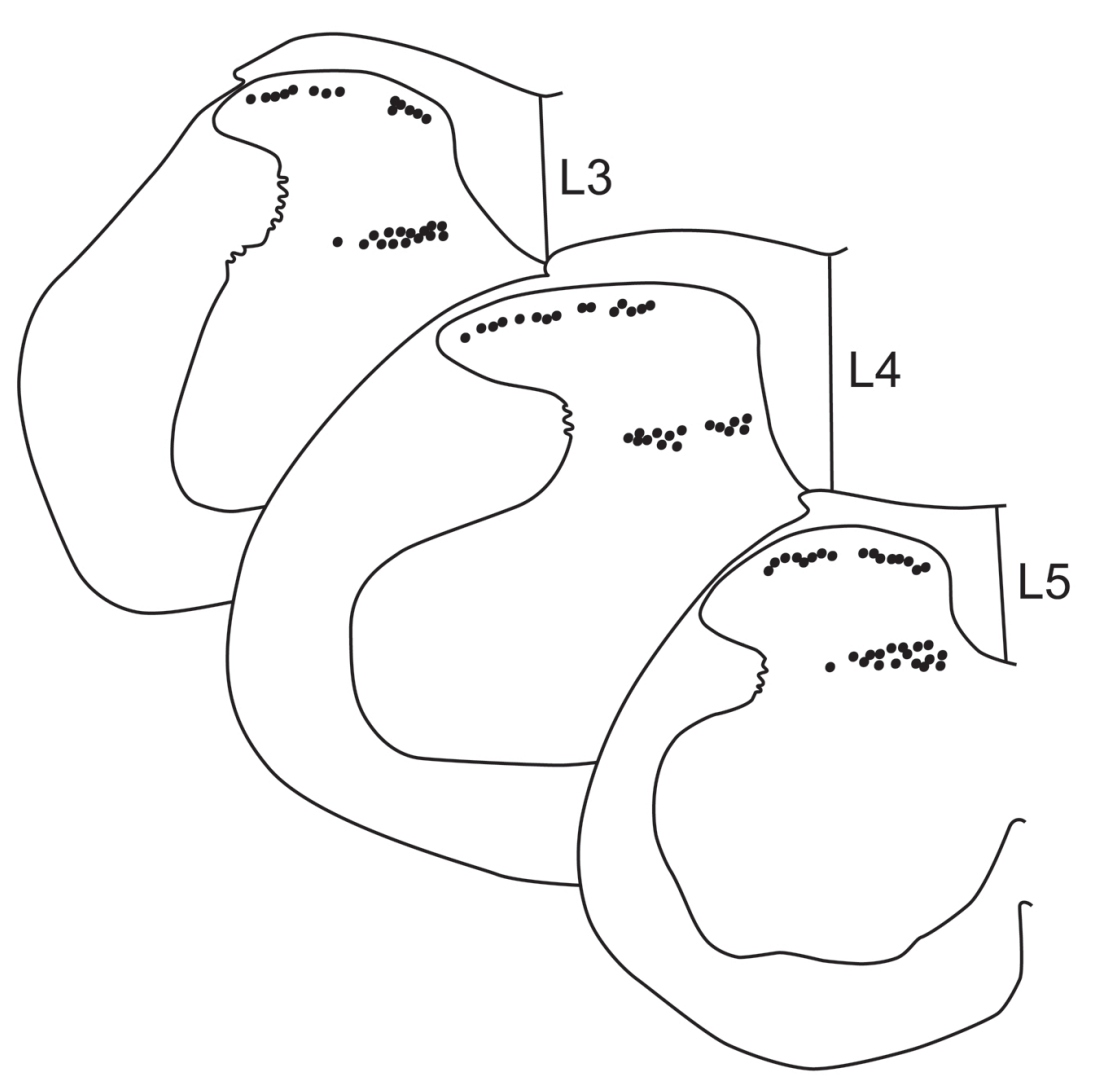
**Location of recorded SDH and DDH neurons in lumbosacral spinal cord**. The location of each recorded neuron was plotted on templates of the L3, L4, and L5 spinal cord segments. Approximately 30 neurons were recorded in each segment. For SDH neurons, recordings were obtained across the entire medio-lateral extent of the dorsal horn. For DDH neurons, recordings were concentrated in the medial two thirds of the dorsal horn because dense myelination impedes visualizing neurons in lateral DDH.

mIPSCs, which are considered to be the postsynaptic response to the spontaneous release of single vesicles of neurotransmitter [34], were recorded as follows. GlyR-mediated mIPSCs were pharmacologically isolated by bath application of the AMPA-kainate receptor antagonist 6-cyano-7-nitroquinoxaline-2,3-dione (CNQX; 10 μM), the GABA_A _receptor antagonist bicuculline (10 μM), and the sodium channel blocker tetrodotoxin (TTX; 1 μM). Data collection commenced 3 minutes after drugs were washed into the recording bath (wash-on) and continued for at least another 3 minutes. These mIPSCs were completely abolished by subsequent bath application of the GlyR antagonist strychnine (1 μM; n = 10). GABA_A_R-mediated mIPSCs were pharmacologically isolated in CNQX (10 μM), strychnine (1 μM), and TTX (1 μM), and were abolished by bath application of bicuculline (10 μM; n = 10). The effect of the synthetic endocannabinoid, methAEA (5 μM), on GlyR- and GABA_A_R -mediated mIPSCs was tested in a subset of neurons where mIPSC frequency was relatively high (> 1 Hz). MethAEA was allowed to wash-on for at least 10 minutes before mIPSCs were recorded for analysis. Each recorded neuron's location was carefully noted and mapped as described previously at the completion of each recording session [35]. The templates for L3, L4, and L5 spinal cord segments (Figure 1) were adapted from Franklin and Paxinos' Mouse Brain Atlas [36].

### Analysis of mIPSC properties

Pharmacologically isolated GlyR- and GABA_A_R -mediated mIPSCs were detected and captured using a sliding template method (semi-automated procedure within Axograph software package [37]). Captured mIPSCs were inspected individually and accepted for analysis when: (1) the captured trace did not contain overlapping mIPSCs; (2) the baseline before the rise, or after the decay phase of the mIPSC trace was stable for > 5 ms; and (3) no time-dependent trend was evident in either mIPSC amplitude or instantaneous frequency over the recording period [38]. Analyses were performed on averaged mIPSCs, obtained by aligning the rising phase of all accepted mIPSCs for a given neuron. Peak amplitude, rise time (calculated over 10-90% of peak amplitude), and decay time constant (calculated over 20-80% of the decay phase) were calculated using semi-automated procedures within Axograph software. In both SDH and DDH neurons, GlyR- and GABA_A_R-mediated mIPSCs were best fit by a single decay time constant [39].

### Analysis of single channel conductance

The single-channel conductance underlying mIPSCs was determined by peak scaled nonstationary noise analysis [40] using the Mini Analysis Program (v6; Synaptosoft, Fort Lee, NJ, USA). This procedure calculates a weighted mean of the underlying multiple conductance states for synaptically located receptors, that is, those generating the recorded mIPSCs versus receptors located outside the synaptic cleft. Briefly, for each neuron, mIPSCs were aligned at the midpoint of their rising phase and averaged. This averaged mIPSC was then scaled to the peak amplitude of all captured mIPSCs that contributed to the averaged mIPSC. The peak scaled average current was then subtracted from individual (scaled) mIPSCs to obtain a difference current, which represents random receptor fluctuations around the mean. Difference currents were binned over the decay phase of the mIPSC. The variance was then plotted against the mean current. A parabolic function (variance = I [current] - [current^2^]/N^P ^+ baseline noise) was then fit to the variance/mean plot, where I is single-channel current, N is the average number of channels open at mIPSC peak, and P is open probability [40].

### RNA extraction and relative real-time PCR

A separate set of experiments were undertaken to quantify Gly-, GABA_A_R and CB_1_R subunit mRNA. Spinal cord slices were prepared as described above and each slice was microdissected into 2 regions containing the SDH and DDH. This procedure yielded two pieces of tissue per slice. The SDH and DDH regions for all slices (six slices, L3 - L5) were pooled by region, and prepared for relative real-time PCR (qPCR). Total RNA was extracted from the tissue using TRIzol reagent (Invitrogen, USA) [41]. Tissue was added to TRIzol reagent and total RNA was prepared according to the manufacturer's instructions. RNA integrity was assessed by A260/A280 ratios (> 1.8), or visualisation of 18s and 28s ribosomal bands by electrophoresis with formaldehyde denaturing 1% agarose gel. Total RNA was treated with DNAse1 (Invitrogen, USA) and reverse transcribed with SuperscriptII reverse transcriptase (Invitrogen, USA) as per manufacturer's instructions. Real-time PCR using SYBR Green PCR Mastermix (PE Applied Biosystems, UK) and an ABI prism 7500 sequence detection system (PE Applied Biosystems, UK) was performed to assess the expression of the GlyR subunit genes (α1-4 and β), GABA_A_R subunit genes (α1-α3,α5, β2-3,γ2) and CB_1_R levels. Primers (Additional File 1) were designed for each gene using Primer Premier 5.0 (Premier Biosoft International, USA). Reactions consisting of 2 × SYBR Green PCR Mastermix, 40 nM of each primer, cDNA template, and nuclease-free water were run in triplicate for each gene on the ABI 7500 sequence detection system under the following conditions: 50°C for 2 min, 95°C for 10 min, 45 cycles of 95°C for 15 s and 60°C for 90 s. Dissociation curves consisting of 95°C for 15 s and 60°C for 15 s, followed by a 2% ramp to 95°C were used to ensure a single product of the correct molecular size was present in each reaction. An average cycle threshold value (Ct) was calculated from triplicate results for each gene. Threshold values were normalized to the housekeeping gene β-actin to provide ΔCt values. Relative expression levels for each gene were then calculated using the formula 2^-ΔCt^. Finally, we calculated a ratio for all GlyR- and GABA_A_R- subunits in a given sample (eg, GlyR α1:α2) and then compared the average ratios between the SDH and DDH.

### Statistical analysis

SPSS v13 software package (SPSS Inc. IL, USA) was used for all statistical analyses. Student's unpaired t-tests were used to compare mIPSC properties recorded in the SDH and DDH, and to compare mIPSC properties before and after exposure to methAEA. One-way ANOVA's compared gene expression data for all GlyR- and GABA_A_R subunits in the SDH and DDH. Student's unpaired t-tests compared gene expression for each subunit (GlyR, GABA_A_R and CB_1_) in the SDH versus DDH. When data were not normally distributed non-parametric statistics (Mann-Whitney two sample tests) were applied. All values are presented as means ± SEM. Statistical significance was set at p < 0.05.

### Drugs

TTX was obtained from Alomone Laboratories (Jerusalem, Israel), and methAEA from Tocris Bioscience (Bristol, UK). All other drugs were purchased from Sigma Chemicals (St Louis, MO, USA).

## Results

Whole-cell patch clamp recordings were obtained from 92 neurons in 18 animals in either the SDH (n = 45) or the DDH (n = 47) as illustrated in Figure 1. Mean animal age (21.4 ± 0.3 vs. 21.9 ± 0.3 days) and series resistance (11.6 ± 0.7 vs. 11.6 ± 0.6 MΩ) were similar for SDH and DDH recordings, suggesting neither age nor recording conditions influenced our results. In contrast, input resistance was higher in SDH neurons (640 ± 65 vs. 260 ± 30 MΩ). This is consistent with morphology data, which shows SDH neurons are smaller than those in the DDH [42,43]. GlyR-mediated mIPSCs were detected in 74% (25/34) of SDH neurons and in almost all DDH neurons (93%; 25/27). In contrast, GABA_A_R-mediated mIPSCs were detected in virtually all neurons in our dorsal horn recordings: 93% (14/15) and 100% (18/18) for SDH and DDH neurons, respectively. Thus, inhibitory synaptic transmission at GlyR-containing synapses is more prominent in the DDH, whereas GABA_A_R containing synapses are distributed similarly in both regions of the mouse spinal cord dorsal horn.

### Glycine receptor-mediated synaptic transmission

**Figure 2:**
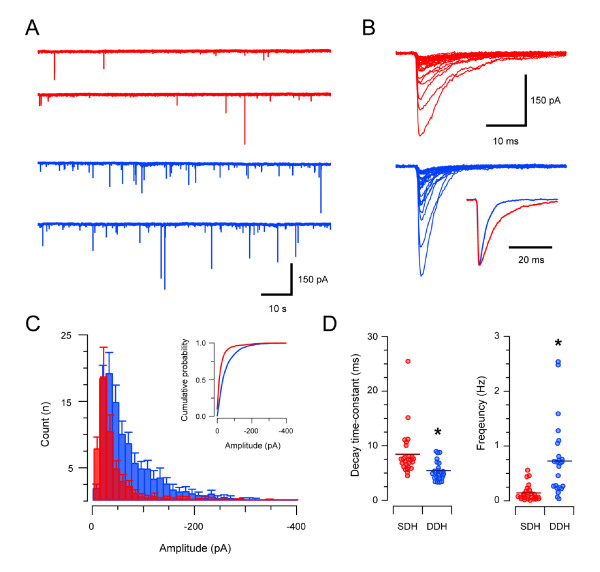
**GlyR-mediated synaptic transmission in the SDH and DDH**. **A **representative traces showing continuous recordings of GlyR-mediated mIPSCs (holding potential - 70 mV) in the presence of TTX (1 μm), CNQX (10 μm), and bicuculline (10 μm) from an SDH (red traces) and a DDH neuron (blue traces). Note mIPSC frequency is considerably higher in DDH neurons. **B **individual mIPSCs from traces in A (aligned at rise onset) showing the amplitude variability in GlyR-mediated mIPSCs recorded in both the SDH (red traces) and DDH (blue traces). Inset shows averaged mIPSCs normalised to the same amplitude (same neurons in A). Note the slower decay time of GlyR-mediated mIPSCs in SDH neurons. **C **overlayed histograms comparing amplitude distributions of GlyR-mediated mIPSCs in SDH (red) and DDH (blue) neurons (n = 25 neurons for SDH and DDH). In the SDH distribution, only 10% of mIPSCs have amplitudes greater than 50 pA, whereas 35% of the mIPSCs in the DDH distribution are greater than 50 pA. Inset shows data presented as cumulative probability plots. **D **plots comparing group data for GlyR-mediated mIPSC decay time-constant and frequency in SDH and DDH neurons. GlyR-mediated mIPSC decay time-constants were slower and mIPSC frequency was lower in SDH neurons.

Figure 2 and Table 1 compare the properties of GlyR-mediated mIPSCs recorded in SDH and DDH neurons. Mean mIPSC amplitude in SDH neurons was approximately half that observed in DDH neurons (37.1 ± 3.9 vs. 64.7 ± 5.0 pA, n = 25 and n = 25 respectively; Figure 2A &2C). mIPSC kinetics also differed between the two regions. The decay time constant for mIPSCs was slower in SDH versus DDH neurons (8.5 ± 0.8 vs. 5.5 ± 0.3 ms), however, mIPSC rise times were identical in the two regions (0.85 ± 0.07 vs. 0.85 ± 0.04 ms). mIPSC frequency was lower in the SDH compared to the DDH (0.15 ± 0.03 vs. 0.72 ± 0.13 Hz). Interestingly, the combined effect of smaller slow decaying mIPSCs in the SDH and larger fast decaying mIPSCs in the DDH resulted in a similar charge transfer per mIPSC in the two regions (364.5 ± 57.7 vs. 456.6 ± 39.9 pA.ms). However, when glycinergic charge is multiplied by mIPSC frequency, to provide an overall measure of glycinergic drive, the result is a more than seven fold greater drive in DDH versus SDH (68.0 ± 26.8 vs. 400.0 ± 107.9 pA.ms.Hz). Of course under in vivo conditions neurotransmitter release is driven largely by action potential invasion of presynaptic terminals. Thus, in vivo differences in background discharge (not assessed in this study) will contribute to overall levels of glycinergic drive in the two regions.

**Table 1:** Properties of mIPSCs in mouse SDH and DDH neurons

*mIPSC properties*								*Channel properties*			
**mIPSC** **type**	**Region**	**Amplitude** **(pA)**	**Rise time** **(ms)**	**Decay time** **(ms)**	**Frequency** **(Hz)**	**Chargeδ** **(pA.ms)**	**n**	**Conductance** **(pS)**	**Open probability** **(P_o_)**	**Channel** **number**	**n**

***Glycine***	**SDH**	37.1 ± 3.9*	0.85 ± 0.07	8.5 ± 0.8*	0.15 ± 0.03*	364.5 ± 57.7	25	54.3 ± 1.6	.97 ± 0.01	10.3 ± 0.5*	8
	**DDH**	64.7 ± 5.0	0.85 ± 0.04	5.5 ± 0.3	0.72 ± 0.13	456.6 ± 39.9	25	55.7 ± 1.8	99 ± 0.01	19.0 ± 5.3	11
***GABA*_*A*_**	**SDH**	25.6 ± 2.4	1.97 ± 0.18*	23.0 ± 3.2*	0.21 ± 0.08	717.3 ± 75.5*	14	22.7 ± 1.7	0.76 ± 0.04*	18.5 ± 1.6*	8
	**DDH**	25.3 ± 2.0	1.49 ± 0.10	18.9 ± 1.6	0.18 ± 0.04	486.3 ± 52.4	18	22.4 ± 2.0	0.94 ± 0.02	12.9 ± 1.0	11

* indicates significant differences for SDH and DDH values

δ mIPSC charge calculated in Axograph using averages for peak amplitude, rise- and decay times

The marked differences in GlyR-mediated mIPSC amplitude in SDH and DDH neurons could be attributable to specific properties of the GlyR, such as differences in single-channel conductance, number of receptors open during quantal release (N_o_), or channel open probability (P_o_). To distinguish between these possibilities, peak-scaled nonstationary noise analysis was undertaken on a subset of mIPSC recordings from SDH and DDH neurons (Table 1). This analysis showed both unitary conductance (54.3 ± 1.6 vs. 55.7 ± 1.8 pS, n = 8 and n = 11, respectively) and P_o _(0.97 ± 0.01 vs. 0.99 ± 0.01), were identical for GlyRs in the SDH and DDH, however, only half the number of channels (10.3 ± 0.5 vs. 19.0 ± 5.3) contributed to mean quantal current in the SDH versus DDH.

### GABA_A _receptor-mediated synaptic transmission

**Figure 3:**
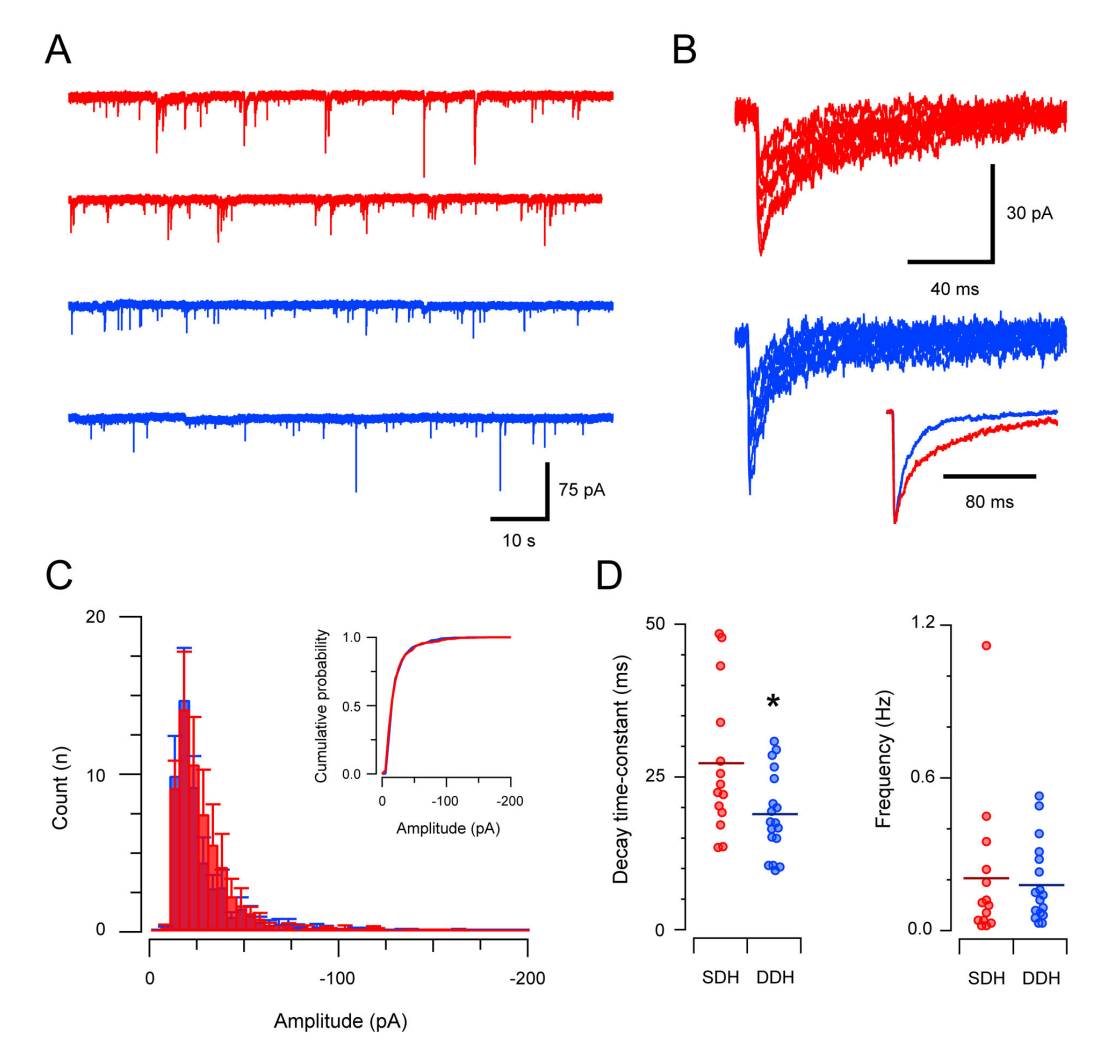
**GABA_A_R-mediated synaptic transmission in the SDH and DDH**. **A **representative traces showing continuous recordings of GABA_A_R-mediated mIPSCs (holding potential - 70 mV) in the presence of TTX (1 μm), CNQX (10 μm), and strychnine (1 μm) from an SDH (red traces) and a DDH neuron (blue traces). Note similar mIPSC frequency in the neurons from the two regions. **B **individual mIPSCs from traces in A (aligned at rise onset) showing amplitude variability of GABA_A_R-mediated mIPSCs in both SDH (red traces) and DDH (blue traces). Inset shows averaged mIPSCs normalised to the same amplitude (same neurons as in A). Note the slower decay time of GABA_A_R-mediated mIPSCs in SDH neurons. **C **overlayed group data histograms comparing amplitude distributions for GABA_A_ergic mIPSCs in SDH (red) and DDH (blue) neurons (n = 14 and 18 for SDH and DDH, respectively). The overlap of the two distributions indicates that GABA_A_R-mediated mIPSC amplitudes are similar in SDH and DDH neurons. Inset shows data presented as cumulative distribution plots. **D **plots comparing group data for GABA_A_R-mediated mIPSC decay time-constant and frequency in SDH and DDH neurons. GABA_A_R-mediated mIPSC decay time-constants were significantly slower in SDH neurons, however, mIPSC frequency was similar in both regions.

Many of the properties of GABA_A_R-mediated mIPSCs were similar in SDH and DDH neurons (Table 1, Figure 3). Specifically, mIPSC amplitude (25.6 ± 2.4 vs. 25.3 ± 2.0 pA, n = 14 and n = 18, respectively) and frequency (0.21 ± 0.08 vs 0.18 ± 0.04 Hz) were similar in both regions (Figure 3A &3C). The rise time and decay time constant of GABA_A_R-mediated mIPSCs, however, was significantly slower in the SDH (1.97 ± 0.18 vs. 1.49 ± 0.10 ms, and 23.0 ± 3.2 vs. 18.9 ± 1.6 ms). Interestingly, the slower decay times of GABA_A_-mediated mIPSCs in the SDH resulted in a significantly greater charge transfer per mIPSC in SDH versus DDH neurons (717.3 ± 75.5 vs. 486.3 ± 52.4 pA.ms). GABA_A_R-mediated drive, as estimated by measuring mIPSC charge transfer by event frequency, however, was similar in both regions (152.4 ± 61.9 vs. 92.8 ± 21.1 pA.ms.Hz).

Peak-scaled nonstationary noise analysis was also applied to GABA_A_R-mediated mIPSCs to examine the properties of synaptically located receptors. This analysis showed that GABA_A_Rs underlying the mIPSCs had an identical unitary conductance in the SDH and DDH (22.7 ± 1.7 vs. 22.4 ± 2.0 pS, n = 8 and n = 11, respectively). P_o _was lower in SDH neurons (0.76 ± 0.04 vs. 0.94 ± 0.02) and N_o _was greater in SDH neurons (18.5 ± 1.6 vs. 12.9 ± 1.0). These data suggest more GABA_A _channels underlie quantal transmission in the SDH but open probability is lower.

### Cannabinoid effects on GlyR-mediated synaptic transmission

**Figure 4:**
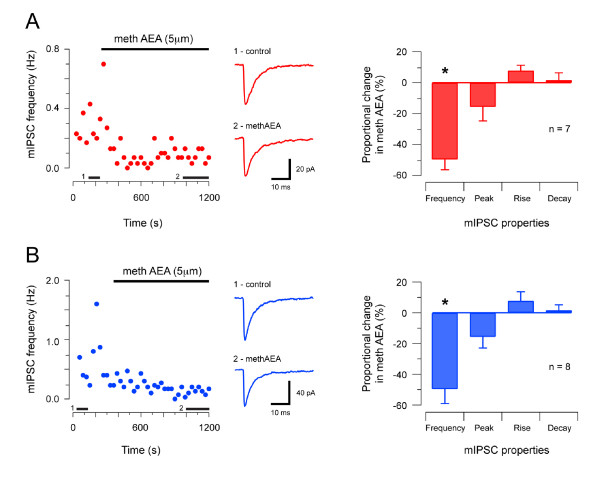
**Effect of methanandamide on GlyR-mediated synaptic transmission in SDH and DDH neurons**. **A **plot showing GlyR-mediated mIPSC frequency (holding potential - 70 mV) in an SDH neuron during bath application of methAEA (5 μm, upper bar). mIPSC frequency declines in the presence of methAEA. Middle traces are averaged mIPSCs (n = 15) under control conditions, and after 10 minutes in methAEA. Black bars above x-axis indicate when averaged mIPSCs were obtained. Plot on right presents group data summarising proportional changes in GlyR-mediated mIPSC properties in methAEA. mIPSC frequency was reduced in methAEA. mIPSC peak amplitude, rise time, and decay time-constant were unaltered. **B **Effect of methAEA on GlyR-mediated mIPSCs in DDH neurons (data presented in same format as A). methAEA also reduced GlyR-mediated mIPSC frequency in DDH neurons without altering mIPSC peak amplitude, rise time, or decay time-constant. Note, each data point in left panels in A and B represent averaged instantaneous mIPSC frequency, binned in 15 s intervals.

Two studies have suggested that cannabinoids have ***direct ***(allosteric) effects on recombinant or cultured GlyRs [31,44]. To test for direct cannabinoid effects on native GlyRs in the SDH or DDH we compared the properties of GlyR-mediated mIPSCs, recorded in both regions, before and after bath application of the synthetic endocannabinoid, methAEA (5 μM). Figure 4A shows the effect of methAEA on glycinergic mIPSCs in the SDH. MethAEA significantly reduced mIPSC frequency (0.18 ± 0.03 vs. 0.08 ± 0.02 Hz, n = 7). In contrast, methAEA had no effect on mIPSC amplitude (35.9 ± 5.7 vs. 31.7 ± 3.4 pA), rise time (0.81 ± 0.16 vs. 0.96 ± 0.08 ms), or decay time constant (7.86 ± 1.10 vs. 7.93 ± 1.03 ms). Figure 4B summarizes the effect of methAEA on GlyR-mediated mIPSCs in the DDH. As in the SDH, methAEA significantly reduced GlyR-mediated mIPSC frequency (0.37 ± 0.09 vs. 0.15 ± 0.03 Hz, n = 8) in DDH neurons. MethAEA, however, did not affect mIPSC amplitude (62.4 ± 6.0 vs. 52.2 ± 6.8 pA), rise time (0.63 ± 0.03 vs. 0.60 ± 0.09 ms), or decay time constant (5.04 ± 0.68 vs. 5.02 ± 0.57 ms). In summary, methAEA reduced mIPSC frequency but had no direct effect on GlyRs located at synapses on either SDH or DDH neurons.

### Cannabinoid effects on GABA_A_R-mediated synaptic transmission

**Figure 5:**
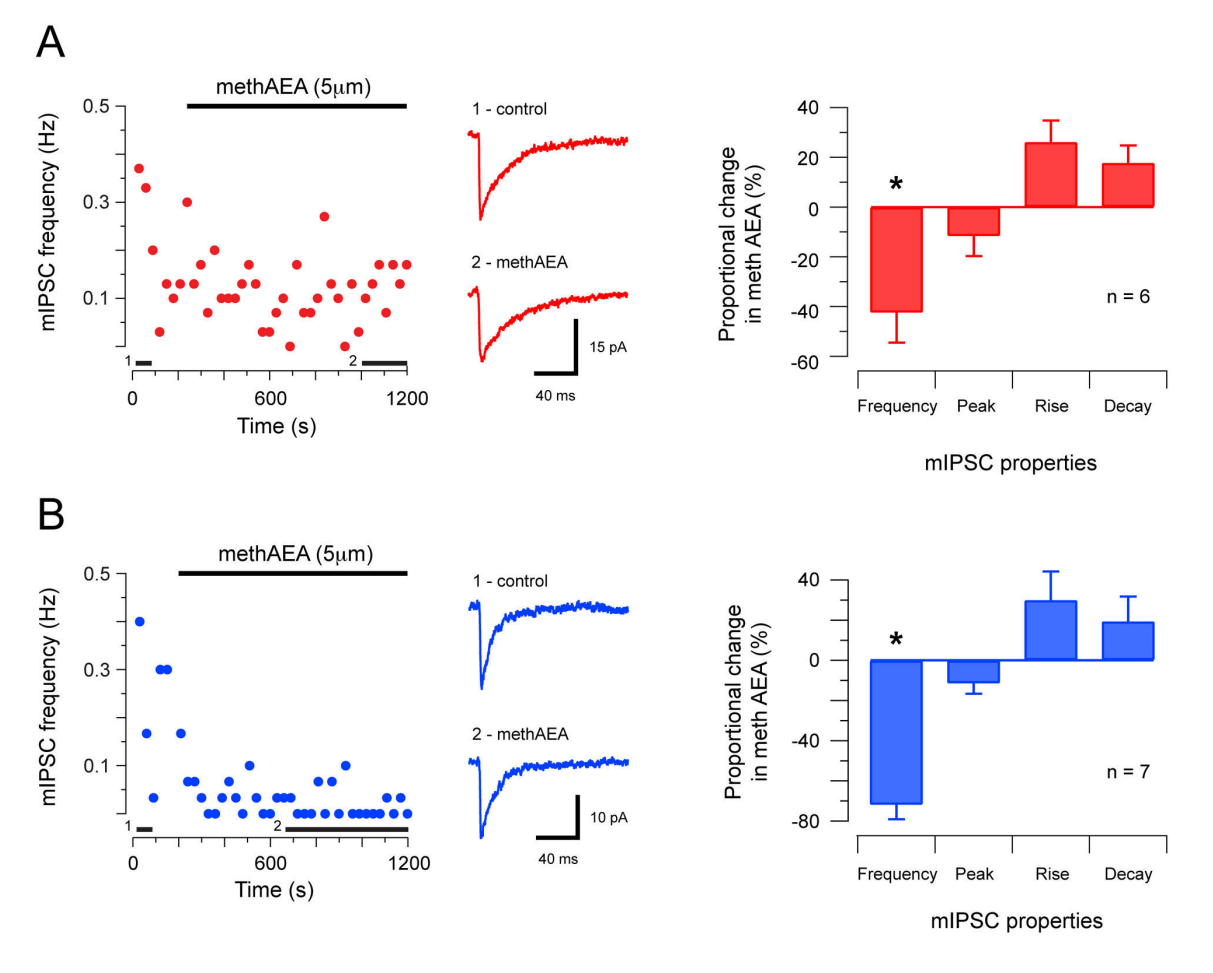
**Effect of methanandamide on GABA_A_R-mediated synaptic transmission in SDH and DDH neurons**. **A **Plot showing GABA_A_R-mediated mIPSC frequency (holding potential - 70 mV) in an SDH neuron during bath application of methAEA (5 μm, upper bar). mIPSC frequency declines significantly in the presence of methAEA. Middle traces are averaged mIPSCs (n = 15) in control conditions and after 10 minutes in methAEA. Black bars above x-axis indicate when averaged mIPSCs were obtained. Plot on right summarizes proportional changes to GABA_A_R-mediated mIPSC properties in methAEA. mIPSC frequency is reduced and rise time is slowed, however, mIPSC peak amplitude and decay time constant are not altered. **B **Effect of methAEA on GABA_A_R-mediated mIPSCs in DDH neurons (presented in same format as A). methAEA significantly reduced GABA_A_R-mediated mIPSC frequency, however, mIPSC peak amplitude, rise time, and decay time-constant were not altered in DDH neurons. Each data point on left panels in A and B represent averaged instantaneous mIPSC frequency, binned in 15 s intervals.

We next tested if methAEA had direct effects on GABA_A_R-mediated mIPSCs in the SDH or DDH. Figure 5A summarises the effect of methAEA on GABA_A_ergic synaptic transmission in SDH neurons. MethAEA produced a significant reduction in mIPSC frequency (0.27 ± 0.05 vs. 0.18 ± 0.03 Hz, n = 6), without altering mIPSC amplitude (30.1 ± 1.7 vs. 28.1 ± 0.9 pA), or decay time constant (29.51 ± 7.39 vs. 37.52 ± 12.57 ms). The rise time of GABA_A_ergic mIPSCs, however, was slowed by methAEA (1.89 ± 0.52 vs. 2.51 ± 0.69 ms). Figure 5B summarises the effect of methAEA on GABA_A_R-mediated mIPSCs in DDH neurons. Bath application of methAEA significantly reduced mIPSC frequency (0.20 ± 0.04 vs. 0.05 ± 0.02 Hz, n = 7), but did not affect mIPSC amplitude (19.6 ± 1.3 vs. 17.0 ± 1.1 pA), rise time (1.58 ± 0.17 vs. 2.10 ± 0.51 ms), or decay time constant (19.70 ± 3.52 vs. 23.10 ± 4.64 ms). These results suggest the predominant effect of methAEA is to reduce GABA_A_R-mediated mIPSC frequency, though the slowed rise times suggest a postsynaptic effect of methAEA, possibly involving a direct interaction on GABA_A_Rs.

### Expression of glycine, GABA_A_, and CB_1 _receptor subunits in SDH and DDH

**Figure 6:**
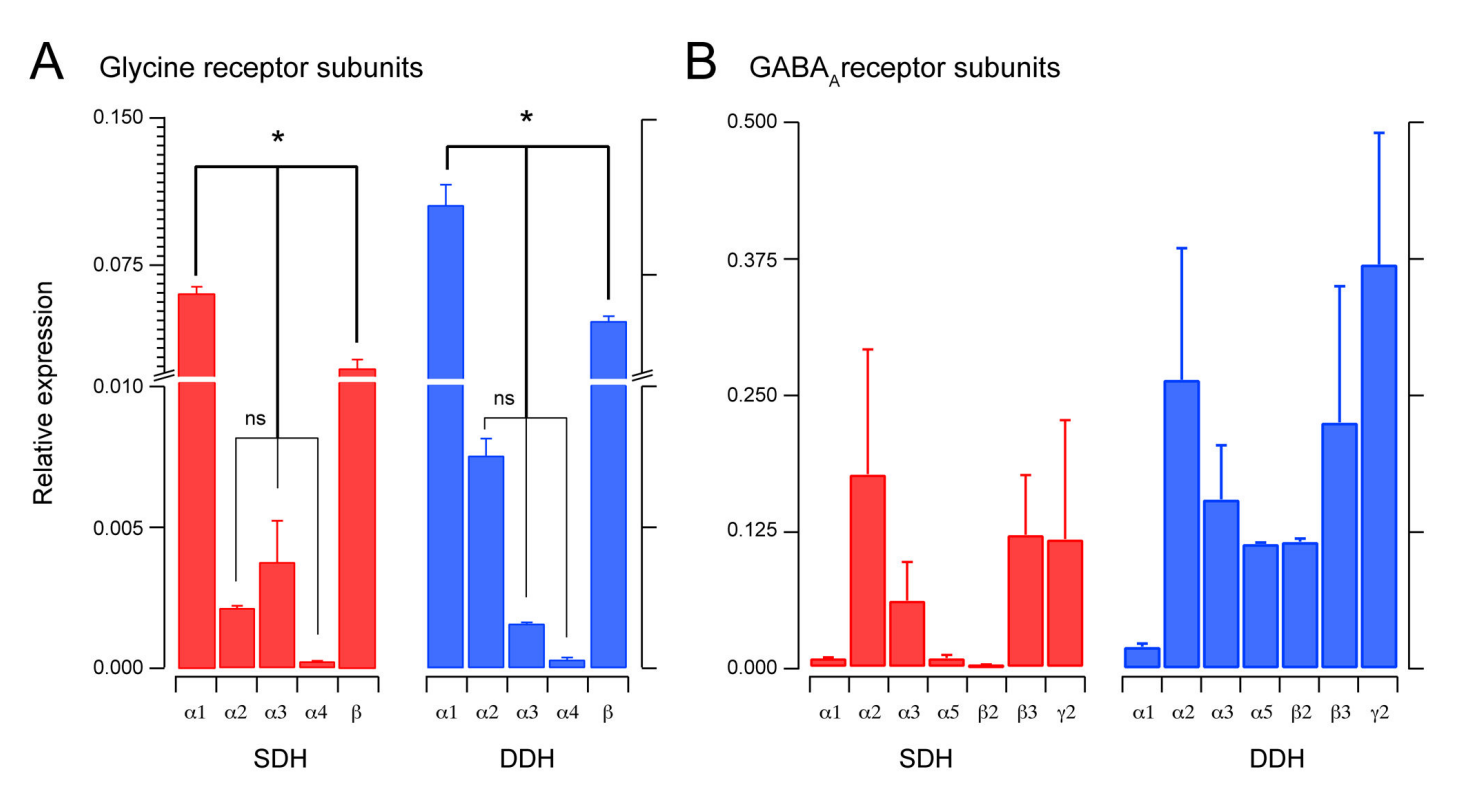
**Gly- and GABA_A_-subunit expression in the SDH and DDH**. **A **plots summarising qPCR analysis for GlyR subunits. Bars represent relative expression of GlyR subunits in the SDH and DDH. The α1 and β subunits were the most highly expressed in both SDH and DDH, whereas expression of the α4 subunit was negligible. The α2 and α3 subunits were expressed at lower levels in both regions. Overall, α1, β, and α2-4 subunits were expressed at significantly different levels in both regions. Note, scale on the y-axis has been broken and expanded to facilitate comparison of subunits showing lower expression levels. **B **plots summarising qPCR analysis for GABA_A_R subunits. Bars represent relative expression of GABA_A_R subunits in the SDH and DDH. Overall, GABA_A_R subunit expression was more variable than that observed for GlyR subunits. No significant differences in expression levels were identified, however, the expression profile for each subunit was similar in both SDH and DDH (ie, higher expression of α2, α3, β3, and γ2).

Because GlyR- and GABA_A_R-mediated mIPSC properties differ markedly in SDH and DDH neurons, we next analysed subunit expression of both receptors in each region using qPCR. Figure 6 summarises qPCR results for Gly- and GABA_A_R subunits in the SDH and DDH. Data are presented as relative expression values. In the SDH (Figure 6A, left) Glyα1 is most highly expressed, followed by Glyβ, Glyα3 and Glyα2, with negligible expression of Glyα4. In the DDH (Figure 6A, right), Glyα1 is again the most highly expressed subunit, followed by Glyβ. Glyα2, Glyα3, and Glyα4 are expressed at much lower levels. Comparison of each GlyR subunit in SDH versus DDH showed Glyα1, Glyα2, Glyα4 and Glyβ were more highly expressed in the DDH (Figure 6A). The results of GABA_A_R subunit expression in the SDH (Figure 6B, left) showed no significant differences in subunit expression. In the DDH (Figure 6B, right), qPCR analysis also failed to resolve any differences in GABA_A_R subunit expression. Comparison of each GABA_A_R subunit in SDH versus DDH shows that only expression levels of GABAα1 and GABAβ2 differed in the two regions. Again higher expression levels were detected in the DDH. Finally, comparison of CB_1_R relative expression using qPCR in the SDH and DDH detected significantly higher relative expression in the DDH (0.007 ± 0.001 vs. 0.025 ± 0.006, respectively).

Because both GlyR and GABA_A_Rs are heteropentamers at native synapses we further analyzed all possible subunit combinations for both receptor types in the SDH and DDH. Comparisons for GlyR subunit combinations are presented in Table 2. This analysis showed possible subunit combinations are differentially weighted in the two regions. For example, the Glyα1:GlyRα2 ratio is higher in the SDH, whereas the Glyα1:GlyRα3 ratio is higher in the DDH. This finding suggests an increased likelihood of Glyα1:GlyRα2 combinations in the DDH and that Glyα1:GlyRα3 combinations would be more prevalent in the SDH. A similar analysis was conducted for GABA_A_R subunits and none of the possible comparisons were significantly different (data not shown).

**Table 2:** Glycine receptor subunit expression ratio's in SDH and DDH

	Glyα1		Glyα2		Glyα3		Glyα4		Glyβ	
	***SDH***	***DDH***	***SDH***	***DDH***	***SDH***	***DDH***	***SDH***	***DDH***	***SDH***	***DDH***

**Glyα1**			0.04 ± 0.00	0.08 ± 0.01	0.06 ± 0.02*	0.02 ± 0.00*	0.00 ± 0.00	0.00 ± 0.00	0.39 ± 0.10	0.51 ± 0.04
**Glyα2**	29.1 ± 2.8 *	12.6 ± 1.1 *			1.8 ± 0.8 *	0.20 ± 0.01 *	0.12 ± 0.00 *	0.05 ± 0.00 *	12.5 ± 0.4 *	7.6 ± 1.3 *
**Glyα3**	21.2 ± 4.2 *	63.2 ± 6.9 *	0.78 ± 0.18 *	5.0 ± 0.2 *			0.11 ± 0.0 *	0.23 ± 0.01 *	18.7 ± 6.6	33.5 ± 2.8
**Glyα4**	252.6 ± 20.8	269.8 ± 22.0	8.7 ± 0.3 *	21.4 ± 0.3 *	9.1 ± 0.3 *	4.3 ± 0.3 *			166.2 ± 53.9	467.3 ± 3.3
**Glyβ**	3.7 ± 1.6	1.5 ± 0.2	0.08 ± 0.00 *	0.11 ± 0.01 *	0.07 ± 0.1	0.03 ± 0.00	0.01 ± 0.00	0.01 ± 0.00		

* indicates significant difference in subunit ratios between SDH and DDH

## Discussion

We found the properties of GlyR- and GABA_A_-mediated mIPSCs to be clearly different in SDH and DDH, suggesting the contribution of each receptor type to fast synaptic inhibition differs between the two regions. An additional aim of our experiments was to examine the action of an endocannabinoid on the two ligand-gated ion channels that are important for inhibitory signalling in the dorsal horn of the spinal cord. Our experiments were driven by three considerations: both Gly- and GABA_A_Rs have been implicated in the onset and maintenance of various pain states; the existence of a unique type of GlyR, containing α3 subunits in the SDH of the mouse spinal cord; and reports of a direct (allosteric) action of cannabinoids on GlyRs in oocytes and dissociated neurons. We found no evidence for a 'direct effect' of methAEA on GlyR function in either the SDH or DDH. The rise time of GABA_A_R-mediated mIPSCs, however, was slowed by methAEA in SDH neurons, suggesting a direct effect on GABA_A_R-mediated mIPSCs. Our real-time PCR data showed the balance of Gly- and GABA_A_R subunit expression differed somewhat in SDH and DDH. For GlyRs, the α1 and β subunits dominate in both SDH and DDH, expression of the α2 GlyR subunit was higher in DDH, whereas α3 subunits where expressed equally in both regions. For GABA_A_Rs, α1 and β2 subunit expression was higher in the DDH. In contrast to previous reports, using immunohistochemistry, we found CB_1 _receptor expression to be higher in the DDH.

### Contributions of glycine- and GABA_A_Rs in dorsal horn function

To our knowledge this study is the first comparison of Gly- and GABA_A_R properties in superficial and deep laminae of the mouse spinal cord dorsal horn. Previously, we have used parasagittal slices (vs. transverse in this study) to compare Gly- and GABA_A_R-mediated mIPSCs in superficial laminae (I-II) of wildtype C57/Bl6 mice and the GlyR mutants *spastic *and *oscillator *[39]. As in the present study, a greater proportion of SDH neurons received GABA_A_R- versus GlyR-mediated inhibition. GABA_A_R - and GlyR-mediated mIPSC amplitude was also similar to that observed in wildtype mice in our previous work. In parasagittal slices, however, mIPSC frequency was higher and decay time constants were slower for both Gly- and GABA_A_Rs. The higher mIPSC frequency is probably due to the rostro-caudal orientation of the dendritic trees of SDH neurons [45] as their parasagittal orientation would result in retention of more synapses in a slice. It is unclear, however, why decay time constants are slower in parasagittal slices.

Our data show that the contribution of GlyRs to fast inhibitory synaptic transmission is greater in the DDH versus SDH, whereas GABA_A_R-mediated inhibition appears to be equally important in both regions. In rat, Cronin et al. (2004) used c-Fos expression to functionally assess tonic inhibitory drive mediated by GlyRs and GABA_A_Rs in dorsal horn and suggested GlyR-mediated mechanisms are more important for setting inhibitory tone in DDH. Our data are consistent with this finding and other data for rats. For example glycine-containing neurons [46,47] and glycine terminals are more concentrated in deeper dorsal horn laminae [48]. In contrast, GABA containing neurons and terminals populate the entire dorsal horn [46,49]. Thus, our mouse data are consistent with a clear regional variation of glycinergic and GABA_A_ergic inhibition in the rodent dorsal horn.

In mouse, Gly- and GABA_A_Rs with differing physiological properties contribute to fast synaptic inhibition in the SDH and DDH. Our data can be compared to a recent study in rat [50], even though the definition of "deep" dorsal horn varied from ours (laminae III-IV vs. laminae IV-VI). In the rat study, a greater proportion of neurons in the DDH received GlyR-mediated mIPSCs and receptor properties were similar in each region. We also found almost all neurons received GlyR-mediated mIPSCs in the mouse DDH, however, the properties of GlyRs differed markedly in SDH versus DDH. Specifically, mIPSC frequency and amplitudes were higher, and mIPSC decay times were faster in the DDH. Developmental processes may explain these differences as the rat study used younger animals (P10-15 rats vs. P17-37 mice). The importance of GABA_A_R- and GlyR- mediated inhibitory processing mechanisms changes in SDH neurons during postnatal development (at least from P0-14), with glycinergic transmission maturing later [51]. Exactly when inhibitory mechanisms are functionally mature in rat is not known. In mice, however, we have shown that GlyR properties do not change in SDH neurons after P17 [39]. Moreover, SDH neurons are certainly electrically mature in the P17-37 mice used in this study [33]. It is unknown whether DDH neurons are mature by P17. Together, this work suggests significantly more GlyRs, with faster kinetics, contribute to GlyR-mediated inhibition in mouse DDH.

GlyRs in mouse SDH and DDH differ in their decay times (8-10 ms vs. 4-5 ms, respectively). The fast decay times of GlyR-mediated mIPSCs in DDH neurons (4-5 ms) match previous reports for both mice [52] and rats [53,54] for GlyRs that contain α1 and β subunits. One potential explanation for the slower kinetics of SDH versus DDH GlyRs in mouse is the existence of a distinctly expressed type of GlyR, containing α3 subunits, in lamina II of the mouse SDH [22]. As for other GlyRs, subunit composition (ie, Glyα1 vs. Glyα2) can shape channel kinetics [53,55]. This, however, does not appear to be the case for α3 containing GlyRs in the mouse SDH as decay times, at least for evoked GlyR-mediated currents, are identical in wildtype and α3 knockout mice [22]. One explanation is that the somato-dendritic distribution of inhibitory synapses may differ for SDH versus DDH neurons. For example, preferential localization of GlyRs on dendrites would decrease the amplitude and slow the decay time of GlyR-mediated mIPSCs [56]. Such dendritic filtering effects would, however, also slow rise times of GlyR-mediated mIPSCs and this was not the case (Table 1). Thus, future experiments are needed to determine why the kinetics of GlyR channels in the SDH and DDH differ.

In contrast to the marked difference in the contribution of GlyRs to inhibition in the SDH and DDH, GABA_A_R-mediated inhibition appears equally important in both spinal cord regions. These observations are consistent with immunohistochemical data in rat showing that GABA-containing neurons, GABA positive terminals and GABA_A_Rs are equally distributed across the dorsal horn. The only major difference we observed in mouse dorsal horn was a slower decay time constant (23 vs. 18 ms) in SDH versus DDH neurons. The faster kinetics of mIPSCs in DDH neurons, are consistent with higher expression of the α1 GABA_A_R subunit in deeper lamina [57] as incorporation of the α1 subunit decreases channel open time and mIPSC decay time [58,59]. Thus, in mice, GABA_A_R-mediated inhibitory transmission appears equally important across the entire dorsal horn, however, GABA_A_Rs in the DDH have faster kinetics.

The different decay times we report for both GlyR- and GABA_A_R-mediated mIPSCs in mouse SDH versus DDH point to varying subunit composition. Our qPCR data for GlyR subunits show that the balance of GlyR-subunit expression differs in SDH and DDH. Not surprisingly, the α1 and β subunits of the GlyR, the ubiquitous adult form of the receptor [60,61], dominate in both regions. The α2 subunit, however, is expressed at higher levels in the DDH. This can not explain the slower kinetics of SDH mIPSCs, as developmental studies in spinal cord [55] and brainstem neurons [39,53] show GlyRs containing α2 subunits have slower kinetics. Interestingly, our qPCR data did not show higher α3 expression in the SDH as reported by Harvey et al., (2004) using immunohistochemistry. A scenario where α3 subunit is preferentially directed to synaptic locations in SDH, whereas in DDH the protein remains at extrasynaptic locations would explain these differences. This needs to considered when comparing electrophysiological data, which only assesses synaptic receptors versus qPCR data which assesses subunit expression without considering location. Our qPCR data for GABA_A_R subunits showed there was significantly greater expression of GABA_A_R α1 and β2 subunits in the DDH versus the SDH. These data are consistent with reports showing GABA_A_Rs containing α1 subunits have faster kinetics [58,59].

### Endocannabinoid actions on fast inhibitory receptors in SDH and DDH

Our data show clearly that the endocannabinoid analogue, methAEA (5 μM), reduces GlyR- and GABA_A_R-mediated mIPSC frequency in both SDH and DDH neurons. These findings are consistent with the "in vivo" view of endcannabinoid action, whereby they are released postsynaptically and act retrogradely at presynaptic terminals to reduce neurotransmitter release [62-66]. The reduced mIPSC frequency we measured in the presence of methAEA is also consistent with immunohistochemical investigations showing that the CB_1_R is expressed on the presynaptic terminals of local circuit neurons, descending inputs, as well as peripheral sensory afferents in the spinal cord [25,62,67]

The negative action of methAEA on both GlyR- and GABA_A_R- mediated inhibition appears to be at odds with the well-documented antinociceptive effects of cannabinoids. In vivo administration of GlyR and GABA_A_R antagonists produces hyperalgesia and tactile allodynia rather than analgesia [68,69]. These apparently conflicting observations made using in vitro and in vivo preparations emphasise that the **net effect **of cannabinoids on spinal circuits determines dorsal horn output. It is well known that cannabinoids also decrease excitatory drive in the dorsal horn, based on reduction of glutamate-mediated mEPSCs [62,70]. New information using paired recording techniques in the SDH indicates that most (~70%) of the connections within lamina II are excitatory [71]. These new data fit with the "net effect" hypothesis. Perhaps these comparisons emphasize the lack of information on specific circuits in spinal cord pain pathway and the roles of various interneuronal populations in dorsal horn function)[72].

Because two recent reports suggest cannabinoids can ***directly ***modulate GlyRs in isolated neurons or oocytes [31,32], we tested the effects of methAEA on GlyR-mediated mIPSCs in both SDH and DDH neurons. We found no evidence for a 'direct effect' of methAEA on GlyR function in either SDH or DDH. There are several explanations for why we did not observe a direct effect of methAEA on GlyRs. First, our study employed a more 'physiologically intact' preparation where factors such as receptor clustering, local glycine concentration and subunit composition would differ markedly. Second, recent reports suggest the direct effects of cannabinoids on recombinant GlyRs is subunit-specific, and glycine-concentration dependent [73].

We also tested for direct effects of cannabinoids on GABA_A_ergic mIPSCs. In both SDH and DDH neurons methAEA reduced mIPSC frequency and did not alter mIPSC amplitude. Similar responses have been reported in the cerebellum [74]. In addition, we observed a significant effect of methAEA on GABA_A_R-mediated mIPSC rise time, however, this was confined to SDH neurons. This is perhaps not surprising, as GABA_A_Rs are modulated by a multitude of exogenous and endogenous substances. For example, the benzodiazepines [75], gaseous and intravenous anaesthetics [76], alcohols [77], neurosteroids [78] and zinc [79] can all positively modulate the GABA_A_R responses to GABA via allosteric actions on the receptor complex. Thus, cannabinoids may prove to be yet another modulatory agent of GABA_A_R-mediated signaling.

### Implications for spinal cord processing of sensory information

Previous work has shown that the SDH and DDH receive different types of peripheral input, project to different supraspinal targets and exhibit considerable variation in their intrinsic connectivity [18,19,71]. We propose that clear differences also exist in inhibitory control mechanisms within each region. Glycinergic signalling dominates in the DDH, whereas GABA_A _signalling is equally important in both regions. Finally, there appears to be need for an inhibitory system with fast and slow kinetics *within *both superficial and deep regions of the mouse spinal cord. GlyR-mediated inhibition is more important in deep regions of the dorsal horn, which preferentially receive peripheral inputs from axons with high conduction velocities [3,12]. The existence of large and fast inhibitory inputs in the DDH would be well suited to modulate the effects of such inputs. In contrast, smaller and slower GABA_A_R-mediated inhibition appears to be equally important in both superficial and deep regions of the spinal cord dorsal horn. These features suggest GABA_A_R-mediated inhibition is more important for fine-tuning the effects of a functionally wider range of peripheral inputs.

Suppression of inhibitory signalling in the dorsal horn, which occurs in certain chronic pain states, can lead to hypersensitivity and tactile allodynia [68,80-82]. Both SDH and DDH neurons have been implicated in this form of plasticity, reinforcing the notion that both regions of the dorsal horn play key roles in nociception even though the SDH preferentially receives nociceptive input. The segregation of excitation and information processing in the two regions is hypothesised to play a key role in the segregation of noxious and innocuous sensory experience. The importance of both glycinergic and GABA_A_ergic inhibition in this segregation was clearly illustrated in recent calcium imaging experiments in spinal cord slices [83]. In the control condition, dorsal root stimulation resulted in discrete and localized excitation that was restricted to the SDH. When the same preparation was stimulated under conditions where inhibition was blocked, excitation spread from the SDH to DDH and even contralaterally. These findings highlight the importance of inhibitory control of cross talk between the SDH and DDH. Consequently, a greater understanding of the properties of inhibitory mechanisms in the two regions will help identify new strategies for treating nociceptive dysfunction in the spinal cord dorsal horn.

## Competing interests

The authors declare that they have no competing interests.

## Authors' contributions

WBA and BAG participated equally in the experimental design, performing experiments, analysis and manuscript preparation. NJB and PAT performed and analyzed the qPCR experiments. The work was carried out in the laboratories of AMB and RJC who contributed to experimental design, analysis and manuscript preparation. All authors have read and approved the final version of the manuscript

## Supplementary Material

Additional file 1 **Primer sequences for PCR analysis**. Table showing primer sequences for PCR analysis. Click here for file

## References

[B1] Willis WD Coggeshall RE Sensory Mechanisms of the Spinal Cord 2004 3 New York: Kluwer Academic/Plenum Publishers

[B2] Todd AJ Koerber HR McMahon SB, Koltzenburg M Neuroanatomical substrates of spinal nociception Wall and Melzack's Textbook of Pain 2006 5 Philadelphia: Elsevier Churchill Livingston 73 90

[B3] Brown AG The dorsal horn of the spinal cord Q J Exp Physiol 1982 67 193 212 6281848 10.1113/expphysiol.1982.sp002630

[B4] Light AR Perl ER Re-examination of the dorsal root projection to the spinal dorsal horn including observations on the differential termination of coarse and fine fibers J Comp Neurol 1979 186 117 131 10.1002/cne.901860202 447880

[B5] Light AR Perl ER Spinal termination of functionally identified primary afferent neurons with slowly conducting myelinated fibers J Comp Neurol 1979 186 133 150 10.1002/cne.901860203 109477

[B6] Christensen BN Perl ER Spinal neurons specifically excited by noxious or thermal stimuli: marginal zone of the dorsal horn J Neurophysiol 1970 33 293 307 5415075 10.1152/jn.1970.33.2.293

[B7] Sugiura Y Lee CL Perl ER Central projections of identified, unmyelinated (C) afferent fibers innervating mammalian skin Science 1986 234 358 361 10.1126/science.3764416 3764416

[B8] Tuckett RP Wei JY Response to an itch-producing substance in cat. I. Cutaneous receptor populations with myelinated axons Brain Res 1987 413 87 94 10.1016/0006-8993(87)90156-9 3594260

[B9] Tuckett RP Wei JY Response to an itch-producing substance in cat. II. Cutaneous receptor populations with unmyelinated axons Brain Res 1987 413 95 103 10.1016/0006-8993(87)90157-0 3594261

[B10] Vallbo AB Olausson H Wessberg J Unmyelinated afferents constitute a second system coding tactile stimuli of the human hairy skin J Neurophysiol 1999 81 2753 2763 10368395 10.1152/jn.1999.81.6.2753

[B11] Koerber HR Woodbury CJ Comprehensive phenotyping of sensory neurons using an ex vivo somatosensory system Physiol Behav 2002 77 589 594 10.1016/S0031-9384(02)00904-6 12527004

[B12] Koerber HR Brown PB Quantitative analysis of dorsal horn cell receptive fields following limited deafferentation J Neurophysiol 1995 74 2065 2076 8592197 10.1152/jn.1995.74.5.2065

[B13] Woolf CJ Central terminations of cutaneous mechanoreceptive afferents in the rat lumbar spinal cord J Comp Neurol 1987 261 105 119 10.1002/cne.902610109 3624538

[B14] Craig ADB Pain mechanisms: labeled lines versus convergence in central processing Ann Rev Neurosci 2003 26 1 30 10.1146/annurev.neuro.26.041002.131022 12651967

[B15] Klop EM Mouton LJ Hulsebosch R Boers J Holstege G In cat four times as many lamina I neurons project to the parabrachial nuclei and twice as many to the periaqueductal gray as to the thalamus Neuroscience 2005 134 189 197 10.1016/j.neuroscience.2005.03.035 15953685

[B16] Kobayashi Y Distribution and morphology of spinothalamic tract neurons in the rat Anat Embryol (Berl) 1998 197 51 67 10.1007/s004290050119 9462858

[B17] Willis WD Jr Zhang X Honda CN Giesler GJ Jr Projections from the marginal zone and deep dorsal horn to the ventrobasal nuclei of the primate thalamus Pain 2001 92 267 276 10.1016/S0304-3959(01)00268-8 11323148

[B18] Lu Y Perl ER A Specific inhibitory pathway between substantia gelatinosa neurons receiving direct C-fiber input J Neurosci 2003 23 8752 8758 14507975 10.1523/JNEUROSCI.23-25-08752.2003 PMC6740424

[B19] Lu Y Perl ER Modular organization of excitatory circuits between neurons of the spinal superficial dorsal horn (laminae I and II) J Neurosci 2005 25 3900 3907 10.1523/JNEUROSCI.0102-05.2005 15829642 PMC6724918

[B20] Schneider SP Local circuit connections between hamster laminae III and IV dorsal horn neurons J Neurophysiol 2008 99 1306 1318 10.1152/jn.00962.2007 18184889

[B21] Cronin JN Bradbury EJ Lidierth M Laminar distribution of GABAA- and glycine-receptor mediated tonic inhibition in the dorsal horn of the rat lumbar spinal cord: effects of picrotoxin and strychnine on expression of Fos-like immunoreactivity Pain 2004 112 156 163 10.1016/j.pain.2004.08.010 15494196

[B22] Harvey RJ Depner UB Wassle H Ahmadi S Heindl C Reinold H Smart TG Harvey K Schutz B Abo-Salem OM Zimmer A Poisbeau P Welzl H Wolfer DP Betz H Zeilhofer HU Muller U GlyR alpha3: an essential target for spinal PGE2-mediated inflammatory pain sensitization Science 2004 304 884 887 10.1126/science.1094925 15131310

[B23] Gilbert PE A comparison of THC, nantradol, nabilone, and morphine in the chronic spinal dog J Clin Pharmacol 1981 21 311S 319S 6271835 10.1002/j.1552-4604.1981.tb02609.x

[B24] Smith PB Martin BR Spinal mechanisms of delta 9-tetrahydrocannabinol-induced analgesia Brain Res 1992 578 8 12 10.1016/0006-8993(92)90222-U 1324767

[B25] Farquhar-Smith WP Egertova M Bradbury EJ McMahon SB Rice AS Elphick MR Cannabinoid CB(1) receptor expression in rat spinal cord Mol Cell Neurosci 2000 15 510 521 10.1006/mcne.2000.0844 10860578

[B26] Richardson JD Aanonsen L Hargreaves KM Hypoactivity of the spinal cannabinoid system results in NMDA-dependent hyperalgesia J Neurosci 1998 18 451 457 9412521 10.1523/JNEUROSCI.18-01-00451.1998 PMC6793401

[B27] Shen M Piser TM Seybold VS Thayer SA Cannabinoid receptor agonists inhibit glutamatergic synaptic transmission in rat hippocampal cultures J Neurosci 1996 16 4322 4334 8699243 10.1523/JNEUROSCI.16-14-04322.1996 PMC6578864

[B28] Lichtman AH Martin BR Cannabinoid-induced antinociception is mediated by a spinal alpha 2-noradrenergic mechanism Brain Res 1991 559 309 314 10.1016/0006-8993(91)90017-P 1665384

[B29] Pugh G Jr Smith PB Dombrowski DS Welch SP The role of endogenous opioids in enhancing the antinociception produced by the combination of delta 9-tetrahydrocannabinol and morphine in the spinal cord J Pharmacol Exp Ther 1996 279 608 616 8930163

[B30] Reche I Fuentes JA Ruiz-Gayo M A role for central cannabinoid and opioid systems in peripheral delta 9-tetrahydrocannabinol-induced analgesia in mice Eur J Pharmacol 1996 301 75 81 10.1016/0014-2999(96)00045-3 8773449

[B31] Hejazi N Zhou C Oz M Sun H Ye JH Zhang L Delta9-tetrahydrocannabinol and endogenous cannabinoid anandamide directly potentiate the function of glycine receptors Mol Pharmacol 2006 69 991 997 16332990 10.1124/mol.105.019174

[B32] Lozovaya N Yatsenko N Beketov A Tsintsadze T Burnashev N Glycine receptors in CNS neurons as a target for nonretrograde action of cannabinoids J Neurosci 2005 25 7499 7506 10.1523/JNEUROSCI.0977-05.2005 16107637 PMC6725404

[B33] Walsh MA Graham BA Brichta AM Callister RJ Evidence for a critical period in the development of excitability and potassium currents in mouse lumbar superficial dorsal horn neurons J Neurophysiol 2009 101 1800 1812 10.1152/jn.90755.2008 19176612

[B34] Bekkers JM Stevens CF NMDA and non-NMDA receptors are co-localized at individual excitatory synapses in cultured rat hippocampus Nature 1989 341 230 233 10.1038/341230a0 2571090

[B35] Graham BA Brichta AM Schofield PR Callister RJ Altered potassium channel function in the superficial dorsal horn of the spastic mouse J Physiol (Lond) 2007 584 121 136 10.1113/jphysiol.2007.138198 17690143 PMC2277054

[B36] Franklin KBJ Paxinos G Mouse Brain in Stereotaxic Coordinates 1997 3 San Deigo: Academic Press

[B37] Clements JD Bekkers JM Detection of spontaneous synaptic events with an optimally scaled template Biophys J 1997 73 220 229 10.1016/S0006-3495(97)78062-7 9199786 PMC1180923

[B38] Callister RJ Walmsley B Amplitude and time course of evoked and spontaneous synaptic currents in rat submandibular ganglion cells J Physiol (Lond) 1996 490 149 157 8745284 10.1113/jphysiol.1996.sp021132 PMC1158653

[B39] Graham BA Schofield PR Sah P Callister RJ Altered inhibitory synaptic transmission in superficial dorsal horn neurones in spastic and oscillator mice J Physiol (Lond) 2003 551 905 916 10.1113/jphysiol.2003.049064 12837931 PMC2343288

[B40] Traynelis SF Silver RA Cull-Candy SG Estimated conductance of glutamate receptor channels activated during EPSCs at the cerebellar mossy fiber-granule cell synapse Neuron 1993 11 279 289 10.1016/0896-6273(93)90184-S 7688973

[B41] Beveridge NJ Tooney PA Carroll AP Gardiner E Bowden N Scott RJ Tran N Dedova I Cairns MJ Dysregulation of miRNA 181b in the temporal cortex in schizophrenia Hum Mol Genet 2008 17 1156 1168 10.1093/hmg/ddn005 18184693

[B42] Molander C Xu Q Grant G The cytoarchitectonic organization of the spinal cord in the rat. I. The lower thoracic and lumbosacral cord J Comp Neurol 1984 230 133 141 10.1002/cne.902300112 6512014

[B43] Rexed B The cytoarchitectonic organization of the spinal cord in the cat J Comp Neurol 1952 96 414 495 10.1002/cne.900960303 14946260

[B44] Lozovaya N Yatsenko N Beketov A Tsintsadze T Burnashev N Glycine Receptors in CNS Neurons as a Target for Nonretrograde Action of Cannabinoids J Neurosci 2005 25 7499 7506 10.1523/JNEUROSCI.0977-05.2005 16107637 PMC6725404

[B45] Chery N Yu XH de Koninck Y Visualization of lamina I of the dorsal horn in live adult rat spinal cord slices J Neurosci Methods 2000 96 133 142 10.1016/S0165-0270(99)00195-8 10720677

[B46] Todd AJ Sullivan AC Light microscope study of the coexistence of GABA-like and glycine-like immunoreactivities in the spinal cord of the rat J Comp Neurol 1990 296 496 505 10.1002/cne.902960312 2358549

[B47] Zeilhofer HU Studler B Arabadzisz D Schweizer C Ahmadi S Layh B Bosl MR Fritschy JM Glycinergic neurons expressing enhanced green fluorescent protein in bacterial artificial chromosome transgenic mice J Comp Neurol 2005 482 123 141 10.1002/cne.20349 15611994

[B48] Zeilhofer HU The glycinergic control of spinal pain processing Cell Mol Life Sci 2005 62 2027 2035 10.1007/s00018-005-5107-2 15968463 PMC11139092

[B49] Mackie M Hughes DI Maxwell DJ Tillakaratne NJ Todd AJ Distribution and colocalisation of glutamate decarboxylase isoforms in the rat spinal cord Neuroscience 2003 119 461 472 10.1016/S0306-4522(03)00174-X 12770560

[B50] Inquimbert P Rodeau JL Schlichter R Differential contribution of GABAergic and glycinergic components to inhibitory synaptic transmission in lamina II and laminae III-IV of the young rat spinal cord Eur J Neurosci 2007 26 2940 2949 10.1111/j.1460-9568.2007.05919.x 18001289

[B51] Baccei ML Fitzgerald M Development of GABAergic and Glycinergic Transmission in the Neonatal Rat Dorsal Horn J Neurosci 2004 24 4749 4757 10.1523/JNEUROSCI.5211-03.2004 15152035 PMC6729459

[B52] Graham BA Schofield PR Sah P Margrie TW Callister RJ Distinct Physiological Mechanisms Underlie Altered Glycinergic Synaptic Transmission in the Murine Mutants spastic, spasmodic, and oscillator J Neurosci 2006 26 4880 4890 10.1523/JNEUROSCI.3991-05.2006 16672662 PMC6674148

[B53] Singer JH Talley EM Bayliss DA Berger AJ Development of glycinergic synaptic transmission to rat brain stem motoneurons J Neurophysiol 1998 80 2608 2620 9819267 10.1152/jn.1998.80.5.2608

[B54] Chery N de Koninck Y Junctional versus extrajunctional glycine and GABA(A) receptor-mediated IPSCs in identified lamina I neurons of the adult rat spinal cord J Neurosci 1999 19 7342 7355 10460241 10.1523/JNEUROSCI.19-17-07342.1999 PMC6782499

[B55] Takahashi T Momiyama A Single-channel currents underlying glycinergic inhibitory postsynaptic responses in spinal neurons Neuron 1991 7 965 969 10.1016/0896-6273(91)90341-V 1722412

[B56] Lim R Alvarez FJ Walmsley B Quantal size is correlated with receptor cluster area at glycinergic synapses in the rat brainstem J Physiol (Lond) 1999 516 Pt 2 505 512 10.1111/j.1469-7793.1999.0505v.x 10087348 PMC2269264

[B57] Bohlhalter S Weinmann O Mohler H Fritschy J Laminar compartmentalization of GABAA-receptor subtypes in the spinal cord: an immunohistochemical study J Neurosci 1996 16 283 297 8613794 10.1523/JNEUROSCI.16-01-00283.1996 PMC6578721

[B58] Bosman LW Heinen K Spijker S Brussaard AB Mice lacking the major adult GABAA receptor subtype have normal number of synapses, but retain juvenile IPSC kinetics until adulthood J Neurophysiol 2005 94 338 346 10.1152/jn.00084.2005 15758057

[B59] Brussaard AB Kits KS Baker RE Willems WP Leyting-Vermeulen JW Voorn P Smit AB Bicknell RJ Herbison AE Plasticity in fast synaptic inhibition of adult oxytocin neurons caused by switch in GABA(A) receptor subunit expression Neuron 1997 19 1103 1114 10.1016/S0896-6273(00)80401-8 9390523

[B60] Legendre P The glycinergic inhibitory synapse Cell Mol Life Sci 2001 58 760 793 10.1007/PL00000899 11437237 PMC11337367

[B61] Lynch JW Molecular structure and function of the glycine receptor chloride channel Physiol Rev 2004 84 1051 1095 10.1152/physrev.00042.2003 15383648

[B62] Morisset V Urban L Cannabinoid-induced presynaptic inhibition of glutamatergic EPSCs in substantia gelatinosa neurons of the rat spinal cord J Neurophysiol 2001 86 40 48 11431486 10.1152/jn.2001.86.1.40

[B63] Twitchell W Brown S Mackie K Cannabinoids inhibit N- and P/Q-type calcium channels in cultured rat hippocampal neurons J Neurophysiol 1997 78 43 50 9242259 10.1152/jn.1997.78.1.43

[B64] Katona I Sperlagh B Sik A Kafalvi A Vizi ES Mackie K Freund TF Presynaptically located CB1 cannabinoid receptors regulate GABA release from axon terminals of specific hippocampal interneurons J Neurosci 1999 19 4544 4558 10341254 10.1523/JNEUROSCI.19-11-04544.1999 PMC6782612

[B65] Schlicker E Kathmann M Modulation of transmitter release via presynaptic cannabinoid receptors Trends Pharmacol Sci 2001 22 565 572 10.1016/S0165-6147(00)01805-8 11698100

[B66] Vaughan CW Christie MJ Retrograde signalling by endocannabinoids Handb Exp Pharmacol 2005 367 383 full_text 16596781 10.1007/3-540-26573-2_12

[B67] Salio C Fischer J Franzoni MF Conrath M Pre- and postsynaptic localizations of the CB1 cannabinoid receptor in the dorsal horn of the rat spinal cord Neuroscience 2002 110 755 764 10.1016/S0306-4522(01)00584-X 11934482

[B68] Yaksh TL Behavioral and autonomic correlates of the tactile evoked allodynia produced by spinal glycine inhibition: effects of modulatory receptor systems and excitatory amino acid antagonists Pain 1989 37 111 123 10.1016/0304-3959(89)90160-7 2542867

[B69] Sivilotti L Woolf CJ The contribution of GABAA and glycine receptors to central sensitization: disinhibition and touch-evoked allodynia in the spinal cord J Neurophysiol 1994 72 169 179 7965003 10.1152/jn.1994.72.1.169

[B70] Jennings EA Vaughan CW Roberts LA Christie MJ The actions of anandamide on rat superficial medullary dorsal horn neurons in vitro J Physiol (Lond) 2003 548 121 129 10.1113/jphysiol.2002.035063 12562891 PMC2342784

[B71] Santos SFA Rebelo S Derkach VA Safronov BV Excitatory interneurons dominate sensory processing in the spinal substantia gelatinosa of rat J Physiol (Lond) 2007 581 241 254 10.1113/jphysiol.2006.126912 17331995 PMC2075233

[B72] Graham BA Brichta AM Callister RJ Moving from an averaged to specific view of spinal cord pain processing circuits J Neurophysiol 2007 98 1057 1063 10.1152/jn.00581.2007 17567772

[B73] Yang Z Aubrey KR Alroy I Harvey RJ Vandenberg RJ Lynch JW Subunit-specific modulation of glycine receptors by cannabinoids and N-arachidonyl-glycine Biochem Pharmacol 2008 76 1014 1023 10.1016/j.bcp.2008.07.037 18755158

[B74] Diana MA Levenes C Mackie K Marty A Short-term retrograde inhibition of GABAergic synaptic currents in rat Purkinje cells is mediated by endogenous cannabinoids J Neurosci 2002 22 200 208 11756503 10.1523/JNEUROSCI.22-01-00200.2002 PMC6757612

[B75] Haefely W Kulcsar A Mohler H Pieri L Polc P Schaffner R Possible involvement of GABA in the central actions of benzodiazepines Adv Biochem Psychopharmacol 1975 131 151 242199

[B76] Lin LH Whiting P Harris RA Molecular determinants of general anesthetic action: role of GABAA receptor structure J Neurochem 1993 60 1548 1553 10.1111/j.1471-4159.1993.tb03320.x 7681105

[B77] Soldo BL Proctor WR Dunwiddie TV Ethanol differentially modulates GABAA receptor-mediated chloride currents in hippocampal, cortical, and septal neurons in rat brain slices Synapse 1994 18 94 103 10.1002/syn.890180204 7839317

[B78] Belelli D Lambert JJ Neurosteroids: endogenous regulators of the GABA(A) receptor Nat Rev Neurosci 2005 6 565 575 10.1038/nrn1703 15959466

[B79] Hosie AM Dunne EL Harvey RJ Smart TG Zinc-mediated inhibition of GABA(A) receptors: discrete binding sites underlie subtype specificity Nat Neurosci 2003 6 362 369 10.1038/nn1030 12640458

[B80] Sivilotti L Woolf CJ The contribution of GABA(A) and glycine receptors to central sensitization: disinhibition and touch-evoked allodynia in the spinal cord J Neurophysiol 1994 72 169 179 7965003 10.1152/jn.1994.72.1.169

[B81] Kontinen VK Stanfa LC Basu A Dickenson AH Electrophysiologic evidence for increased endogenous gabaergic but not glycinergic inhibitory tone in the rat spinal nerve ligation model of neuropathy Anesthesiology 2001 94 333 339 10.1097/00000542-200102000-00024 11176099

[B82] Moore KA Kohno T Karchewski LA Scholz J Baba H Woolf CJ Partial peripheral nerve injury promotes a selective loss of GABAergic inhibition in the superficial dorsal horn of the spinal cord J Neurosci 2002 22 6724 6731 12151551 10.1523/JNEUROSCI.22-15-06724.2002 PMC6758148

[B83] Ruscheweyh R Sandkuhler J Long-range oscillatory Ca2+ waves in rat spinal dorsal horn Eur J Neurosci 2005 22 1967 1976 10.1111/j.1460-9568.2005.04393.x 16262635

