# Supplementary material for: Different forms of glycine- and GABAA-receptor mediated inhibitory synaptic transmission in mouse superficial and deep dorsal horn neurons
Source: Mol Pain. 2009 Nov 18;5:65. doi: 10.1186/1744-8069-5-65 (PMC2784755; doi:10.1186/1744-8069-5-65)
Supplement: Additional file 1 — Primer sequences for PCR analysis. Table showing primer sequences for PCR analysis. [file 1744-8069-5-65-S1.DOC]

## Additional File 1

## Primer sequences for PCR analysis

| **Receptor Subunit** | **Real-time PCR Primer Sequence** |
| --- | --- |
| Glycine α1 FWD | ^5’^CAACAGTTTCGGTTCCATC^3’^ |
| Glycine α1 REV | ^5’^CGCCTCTTCCTCCTAAATCGAAGCAGT^3’^ |
| Glycine α2 FWD | ^5’^GGGACAAACCACTTCAGGAGGC^3’^ |
| Glycine α2 REV | ^5’^TAGCATCTGCATCTTTGGGGGGT^3’^ |
| Glycine α3 FWD | ^5’^GATTTTACTTCTGGGAAGCCGC^3’^ |
| Glycine α3 REV | ^5’^ATAGCTGATTCCCGGTTCAC^3’^ |
| Glycine α4 FWD | ^5’^GGTGTCCTACGTAAAGGCAATT^3’^ |
| Glycine α4 REV | ^5’^CTCCATACGCTGACGTCTCT^3’^ |
| Glycine β FWD | ^5’^GGATCCATTCAAGAGACA^3’^ |
| Glycine β REV | ^5’^AGCCACACATCCAGTGCCTT^3’^ |
| GABA_A_ α1 FWD | ^5’^TGCTGGACGGTTATGACAAT^3’^ |
| GABA_A_ α1 REV | ^5’^GAAACTGGTCCGAAACTGGT^3’^ |
| GABA_A_ α2 FWD | ^5’^ACAACCTTGAGCATCAGTGC^3’^ |
| GABA_A_ α2 REV | ^5’^AATTCACGGTTGCAAATTCA^3’^ |
| GABA_A_ α3 FWD | ^5’^GACAGTCCTGCTGAGACCAA^3’^ |
| GABA_A_ α3 REV | ^5’^ATAGCTGATTCCCGGTTCAC^3’^ |
| GABA_A_ α5 FWD | ^5’^TCCATTGCACACAACATGAC^3’^ |
| GABA_A_ α5 REV | ^5’^GCAGAGATTGTCAGACGCAT^3’^ |
| GABA_A_ β2 FWD | ^5’^AGCTGCTAATGCCAACAATG^3’^ |
| GABA_A_ β2 REV | ^5’^GTCCCATTACTGCTTCGGAT^3’^ |
| GABA_A_ β3 FWD | ^5’^CAAAGCCATCGACATGTACC^3’^ |
| GABA_A_ β3 REV | ^5^’CTTCTCCGCAAGCTTCTTCT^3’^ |
| GABA_A_ γ2 FWD | ^5’^TGGTCACCGAATGTGTTTCT^3’^ |
| GABA_A_ γ2 REV | ^5’^TACTTTGCCTTGCAGGTTTG^3’^ |
| CB_1_ FWD | ^5’^GCTTATCAAGACGGTGTTTGC^3’^ |
| CB_1_ REV | ^5’^GCATGTCTCAGGTCCTTGCT^3’^ |
| β-actin FWD | ^5’^TGAGACCTTCAACACCCCAG^3’^ |
| β-actin REV | ^5’^CATCTGCTGGAAGGTGGACA^3’^ |
